# Chemical Composition and Effect against Skin Alterations of Bioactive Extracts Obtained by the Hydrodistillation of *Eucalyptus globulus* Leaves

**DOI:** 10.3390/pharmaceutics14030561

**Published:** 2022-03-03

**Authors:** Patrícia Moreira, Fábio Jesus Sousa, Patrícia Matos, Gonçalo Sousa Brites, Maria José Gonçalves, Carlos Cavaleiro, Artur Figueirinha, Lígia Salgueiro, Maria Teresa Batista, Pedro Costa Branco, Maria Teresa Cruz, Cláudia Fragão Pereira

**Affiliations:** 1 CNC—Center for Neuroscience and Cell Biology, CIBB—Center for Innovative Biomedicine and Biotechnology, University of Coimbra, 3004-504 Coimbra, Portugal; patriciaraquel_jm@hotmail.com (P.M.); fabio.fdti@hotmail.com (F.J.S.); g.sousabrites3@gmail.com (G.S.B.); trosete@ff.uc.pt (M.T.C.); 2 Faculty of Pharmacy, University of Coimbra, 3000-548 Coimbra, Portugal; patricia_matos_20@hotmail.com (P.M.); mpinho@ff.uc.pt (M.J.G.); cavaleir@ff.uc.pt (C.C.); amfigueirinha@ff.uc.pt (A.F.); ligia@ff.uc.pt (L.S.); 3 LAQV/REQUIMTE—Associated Laboratory for Green Chemistry, Network of Chemistry and Technology, University of Coimbra, 3000-548 Coimbra, Portugal; 4 CIEPQPF, Research Center for Chemical Processes Engineering and Forest Products, Faculty of Pharmacy, University of Coimbra, 3000-548 Coimbra, Portugal; mtpmb@ff.uc.pt; 5 Toxfinder Lda, 3030-199 Coimbra, Portugal; 6 RAIZ—Forest and Paper Research Institute, Eixo, 3800-783 Aveiro, Portugal; pedro.costa.branco@thenavigatorcompany.com; 7 Faculty of Medicine, University of Coimbra, 3000-548 Coimbra, Portugal

**Keywords:** *Eucalyptus globulus*, essential oil, aqueous phenolic extract, skin health, cytotoxicity, inflammation, aging, allergy, hyperpigmentation

## Abstract

*Eucalyptus globulus* is planted extensively for pulp, paper and wood production. Although bioactive compounds obtained from its biomass are used as cosmetics ingredients, the skin effects were not yet fully explored. In order to fill this gap, this work aimed to study the protective effect against skin damage provided by the essential oil (EO) obtained from the hydrodistillation of *Eucalyptus globulus* leaves, and by an extract obtained from the hydrodistillation residual water (HRW). The major compound identified in the EO was 1,8-Cineole, and the phenolic acids in the HRW included gallic acid as the main phenolic constituent. Moreover, non-toxic EO and HRW concentrations were shown to have anti-aging skin effects in vitro, decreasing age-related senescence markers, namely β-galactosidase and matrix metalloproteinases activation, as well as collagen type 1 upregulation. In addition, EO and HRW were found to exhibit depigmenting effects by inhibiting tyrosinase and melanin production, along with potent anti-inflammatory properties. Furthermore, the absence of skin irritation and sensitization in cells exposed to EO and HRW revealed the safety of both extracts for topical use. Taken together, these results highlight the beneficial effects of extracts obtained from *Eucalyptus globulus* biomass for skin aesthetic and health purposes, which should be explored deeply for the prediction of future pharmaceutical and dermocosmetics industrial applications.

## 1. Introduction

The skin is the largest organ of the human body, and is responsible for protection against mechanical and chemical injuries, vitamin D production, thermoregulation, and dehydration prevention; it also supplies innate and adaptive immune defenses and functions as a sensory organ [1]. There are many described skin modifications that are mostly caused by bacteria, fungi or virus exposure, contact with allergens or irritants, or genetic and lifestyle factors (e.g., ultraviolet radiation, smoking, pollution and poor nutrition), but that can also be triggered by immune system problems, diseases, by natural aging. The most common age-related skin conditions which result from structural and functional alterations include benign and malignant tumors, ulcers, dryness, increased pigmentation, the loss of elasticity, and wrinkling, among others [2]. Therefore, with the growing search for health and wellbeing at all ages, which also includes skin health and beauty, the development of skincare products and cosmetics incorporating bioactive compounds to prevent, delay or attenuate skin aging and its external signs has increased exponentially over the last few decades. In accordance with this, the search for innovative products by the industry has stimulated the development of cosmetics with incorporated bioactive compounds. Plant extracts are a rich source of natural bioactive compounds, such as terpenoids and polyphenols, that are already used as ingredients in cosmetics, namely for skin anti-aging purposes due to antioxidant, anti-inflammatory and antimicrobial activities, or tyrosinase and metalloproteinase inhibition effects; the bioactive compounds are very promising as rich sources of ingredients for the development of sustainable and environmentally friendly skincare products [3,4]. 

*Eucalyptus globulus* Labill. is a tree native to Australia which is extensively planted in many countries of Europe, including Portugal [5]. The exploitation of eucalyptus by the pulp, paper and wood industry generates large quantities of residual biomass (bark, leaves and branches), the valorization of which could represent a significant contribution to the circular economy. The potential of *E. globulus* byproducts as a source of bioactive compounds—namely phenolic compounds like phenolic acids, flavonoids or hydrolysable tannins—has already been demonstrated. In fact, *E. globulus* leaves are traditionally used for the treatment of respiratory problems. Furthermore, recent studies highlighted the anti-microbial, antifungal, analgesic and even anticancer properties of extracts and essential oils (EOs) from leaves, which are associated with the reported anti-inflammatory and antioxidant activities [6,7]. Indeed, previous studies have shown that EOs from *E. globulus* leaves possess antioxidant properties demonstrated by their free radical scavenging capacity and activation of antioxidant enzymes, as well as their anti-inflammatory activity, as these inhibit lipoxygenase and reduce nitric oxide (NO) levels [8,9]. The antioxidant and anti-inflammatory activities of other extracts obtained from *E. globulus* leaves have also been described in the literature, namely ethanolic and methanolic extracts, which were found to reduce the levels of NO and of pro-inflammatory mediators [10,11,12], and an aqueous extract that was shown to exhibit free radical scavenging capacity [13]. Increased levels of pro-inflammatory mediators and reactive oxygen species have been shown to play an important role in skin aging [14], and a recent study demonstrated that an ethanolic extract from dried commercial *E. globulus* biomass is able to protect against UV-induced photoaging, reducing wrinkle formation and skin dryness [15]. These relevant findings strongly support the exploration of the potentialities of extracts obtained from *E. globulus* biomass as sources of bioactive ingredients to be used in dermatological formulations for anti-aging skincare. 

In this context, the aim of this study was to explore the protective role against skin damaging insults, namely triggers of senescence, mechanical injury, pigmentation and inflammation, of preparations obtained from fresh *E. globulus* leaves by hydrodistillation, specifically the corresponding EO and the extract that results from the interactions and mass transfer from the plant material to the water which was used in the hydrodistillation process. In addition, the antifungal effect of both extracts was evaluated. To the best of our knowledge, this extract—which we named hydrodistillation residual water extract (HRW)—has never been studied with regard to its chemical composition or bioactivity. Findings from the present study support the use of *E. globulus* leaves as a valuable source of promising bioactive extracts to be incorporated in products for anti-aging skincare.

## 2. Materials and Methods

### 2.1. Plant Material and Extraction

The leaves of 6-years old *E. globulus* trees were collected randomly during October 2018, in a property of The Navigator Company, located at Braçal (GPS coordinates 40°44′5.388 N/8°23′53.97 W), in the region of Sever do Vouga, Portugal. The fresh leaves were submitted to hydrodistillation using a modified Clevenger apparatus as described in the European Pharmacopoeia, in order to isolate the EO. Briefly, the plant material was roughly divided (150 g), and was placed in a round bottom flask with an added 1.5 L distilled water. The modified Clevenger apparatus was adapted, and the distillation was maintained for 3 h with the continuous coobation of the distillation water. The isolated EO was stored in dark vials at 4 °C for further assays. Thereafter, the hydrodistillation residual water, usually discarded, was collected, concentrated in a rotavapor, frozen and freeze-dried. This HRW extract was kept at −20 °C in the dark until use.

### 2.2. Chemical Characterization

The chemical analysis of the EO was performed by a combination of gas chromatography (GC-FID) and gas chromatography coupled to mass spectrometry (GC-MS). The GC-FID analysis was performed with a Hewlett Packard 6890 gas chromatograph (Agilent Technologies, Palo Alto, CA, USA) and a HP GC ChemStation Rev. A.05.04 data handling system equipped with a single injector and two flame ionization detectors (FID). A graphpak divider (Agilent Technologies, Palo Alto, CA, USA, part number 5021-7148) was used for simultaneous sampling on two Supelco (Supelco Inc., Bellefonte, PA, USA) fused silica capillary columns with different stationary phases: SPB-1 (polydimethylsiloxane; 30 m × 0.20 mm i.d., film thickness 0.20 μm) and SupelcoWax-10 (polyethylene glycol; 30 m × 0.20 mm i.d., film thickness 0.20 μm). The carrier gas was helium, which was adjusted to a linear velocity of 30 cm/s. The initial oven temperature was 70 °C, rising to 220 °C at 3 °C/min, and were held at 220 °C for 15 min. The injector and detectors were set to 250 °C. The EO samples were diluted in *n*-pentane (1:10) and injected (0.2 µL) in split mode (1:40). The GC-MS analysis was carried out on a Hewlett Packard 6890 gas chromatograph fitted with a HP1 fused silica column (polydimethylsiloxane; 30 m × 0.25 mm i.d., film thickness 0.25 μm), coupled with a Hewlett Packard Mass Selective Detector 5973 (Agilent Technologies, Palo Alto, CA, USA) operated by HP Enhanced ChemStation software, version A.03.00. The GC parameters were as described above. Additionally, the temperatures were set as follows: interface, 250 °C; MS source, 230 °C; and MS quadrupole, 150 °C. The electron impact energy was 70 eV, the ionization current was 60 µA, and the scan range was 35–350 units, with 4.51 scans/s. The EO components were identified considering, concurrently, (1) the acquired retention indices on both SPB-1 and SupelcoWax-10 columns determined by linear interpolation relative to the retention of a series of *n*-alkanes (C_8_-C_23_), and compared with reference values from authentic products available in the laboratory database of the Faculty of Pharmacy of the University of Coimbra, Portugal and literature data [16]; and (2) the acquired mass spectra compared with reference data from the laboratory database, the Wiley/NIST library [17], and the literature [18]. The relative amount of each component was estimated from the GC peak areas without any correction regarding FID responses.

The analysis of the HRW was performed by high-performance liquid chromatography with a photodiode array detector coupled to mass spectrometry with electrospray ionization (HPLC-PDA-ESI/MS^n^). This analysis was performed in a Surveyor liquid chromatograph equipped with a PDA detector, and in interface with a Finnigan LCQ Advantage Ion Max mass spectrometer (Thermo Fisher Scientific, Waltham, MA, USA) equipped with an ESI ionization chamber. Chromatographic separation was performed at 20 °C on a C18 Spherisorb ODS-2 reverse phase column (150 × 2.1 mm; particle size 3 μm; Waters Corp., Milford, MA, USA) preceded by a guard column Spherisorb ODS- 2 C18 (10 × 4.6 mm; particle size 5 μm; Waters Corp., Milford, MA, USA). The mobile phase consisted of an aqueous solution of 2% formic acid (solvent A) and methanol (solvent B). The gradient profile used was 5–15% (0–10 min), 15–30% (10–15 min), 30–40% (15–45 min), 40–50% (45–60 min), 50–80% (60–70 min) and 80–100% (70–80 min) of solvent B at a flow rate of 200 μL/min. The first detection was made with a PDA detector using 280 and 320 nm as the preferred wavelengths, and the second detection was made in the mass spectrometer. Mass analysis was operated in the negative mode, and was programmed to perform a series of three scans: a full mass (MS) and an MS^2^ and MS^3^ of the most abundant ion. The collision gas was helium, with a normalized collision energy of 35%. Nitrogen was used as the nebulizing gas, with a sheath gas flow of 40 arbitrary unit (au) and an auxiliary gas flow of 5 au. The capillary temperature and voltage were set at 275 °C and −35 V, respectively, and the source voltage was set at 5 kV. The data treatment was carried ot with XCALIBUR software (Thermo Fisher Scientific, Waltham, MA, USA).

### 2.3. Cell Culture

The human keratinocytes (HaCaT, CLS 300493, Eppelheim, Germany), mouse fibroblasts (NIH/3T3, ATCC CRL-1658, Manassas, VA, USA) and mouse melanoma (B-16V, DSMZ ACC-370, Braunschweig, Germany) cell lines were cultured with Dulbecco’s Modified Eagle’s Medium (DMEM) (Sigma-Aldrich, St. Louis, MO, USA), supplemented with 10% (*v/v*) heat-inactivated fetal bovine serum (FBS), 1% (*v/v*) antibiotic solution (from a 10,000 U/mL penicillin and 10,000 µg/mL streptomycin stock) (Gibco, Carlsbad, CA, USA), 3.7 g/L sodium bicarbonate, and 1 mM sodium pyruvate (Sigma-Aldrich, St. Louis, MO, USA). The mouse leukaemic macrophage cell line (RAW 264.7, ATCC TIB-71, Manassas, VA, USA) was cultured in DMEM supplemented with 10% (*v/v*) non-inactivated FBS, 1% (*v/v*) antibiotic solution, 1.5 g/L sodium bicarbonate, and 1 mM sodium pyruvate. The human monocyte (THP-1, ATCC TIB-202, Manassas, VA, USA) cell line was cultured and maintained at a cell density between 2 × 10^5^ and 1 × 10^6^ cells/mL in RPMI 1640 supplemented with 10% (*v/v*) inactivated FBS, 1% (*v/v*) antibiotic solution, 1.5 g/L sodium bicarbonate, 25 mM glucose, 10 mM HEPES, and 1 mM sodium pyruvate. The cells were cultured in 75 cm^2^ flasks and maintained in a humidified 5% CO_2_-95% air atmosphere at 37 °C, and the medium was changed every 2–3 days. The adherent cultures of keratinocytes, fibroblasts and melanocytes were detached with Trypsin-Ethylenediamine tetraacetic acid (EDTA) solution 1X (Sigma-Aldrich, St. Louis, MO, USA) when the cells reached 70–80% confluence, while the macrophages were mechanically detached with a cell scraper, for passage and sub-culturing. The adherent cells were sub-cultured over a maximum of ten passages, and the monocyte suspensions were maintained in the culture for a maximum of 2 months. 

### 2.4. Cell Viability

For the evaluation of the cell viability, a 3-(4,5-dimethylthiazol-2-yl)-2,5-diphenyltetrazolium bromide (MTT) reduction assay was performed. RAW 264.7, B-16V, HaCaT and NIH/3T3 cells were seeded in 96-well plates at densities of 9.6 × 10^4^, 3 × 10^4^, 2 × 10^4^ or 1 × 10^4^ cells/well, respectively, and were allowed to adhere for 24 h. On the day of the experiment, the culture medium was replaced by freshly prepared exposure medium [DMEM supplemented with 1% (*v/v*) FBS]. Concentration–response curves were obtained by incubating the cells for 24 h at 37 °C with 0–1.25 mg/mL EO or 0–100 µg/mL HRW, added from stock solutions prepared in dimethyl sulphoxide (DMSO) or phosphate-buffered saline (PBS), respectively, stored at −20 °C. Each plate also included a solvent control [0.2% (*v/v*) DMSO or 1% (*v/v*) PBS prepared in exposure medium]. After the incubation period, the medium was removed and a fresh solution of MTT (0.5 mg/mL) (Sigma-Aldrich, St. Louis, MO, USA) prepared in Krebs medium (140 mM NaCl, 5 mM KCl, 1 mM NaH_2_PO_4_, 1 mM MgCl_2_, 9.6 mM Glucose, 20 mM HEPES, 1.5 mM CaCl_2_, pH 7.4) was added. The different cell lines were incubated with MTT at 37 °C for 30 min (RAW 264. 7 and B-16V cells), 2 h (HaCaT cells), or 4 h (NIH/3T3 cells). Then, the MTT solution was removed, and the formed formazan crystals were dissolved with DMSO. After 10 min of shaking, the absorbance was measured at 570 nm using a SpectraMax Plus 384 Spectrophotometer (Molecular Devices, San Jose, CA, USA). The results of at least three independent experiments performed in triplicate were expressed as a percentage (%) of the absorbance value obtained in the control, which was considered to be 100%; these were graphically presented as the percentage of cell viability versus the concentration of extracts.

### 2.5. Anti-Inflammatory Effect

#### 2.5.1. Measurement of the Nitric Oxide Production

RAW 264.7 cells were seeded in 96-well plates at a density of 9.6 × 10^4^ cells/well, and were allowed to stabilize for 24 h. Then, the cells were incubated in exposure medium (control) or stimulated with 1 μg/mL lipopolysaccharide (LPS) in the absence or in the presence of 0.32 mg/mL EO or 12.5 μg/mL HRW, for 24 h. The production of NO was evaluated by a colorimetric assay, which measured the secretion of its stable metabolite nitrite using the Griess reagent. Briefly, the cellular supernatants were added to equal volumes of the Griess reagent [0.1% (*w/v*) *N*-(1-naphthyl)-ethylenediamine dihydrochloride and 1% (*w/v*) sulphanilamide containing 5% (*w/v*) H_3_PO_4_], and were incubated in the dark at room temperature (RT) for 30 min. The absorbance at 550 nm was measured with a SpectraMax Plus 384 Spectrophotometer, using the culture medium as a blank. The results of at least three independent experiments performed in triplicate were expressed as % of nitrites produced by cells cultured in the presence of the pro-inflammatory stimulus LPS, which was considered to be 100%.

#### 2.5.2. Expression of Pro-Inflammatory Mediators

RAW 264.7 cells were seeded in 6-well plates at a density of 2.8 × 10^6^ cells/well, and were allowed to stabilize for 24 h. After this, the cells were pre-incubated for 18 h with exposure medium (control), 0.32 mg/mL EO, or 12.5 μg/mL HRW, before incubation with or without 1 μg/mL LPS for an additional 6 h. The RNA was extracted with NZYol reagent (Nzytech, Lisbon, Portugal), the concentration was measured using a NanoDrop 2000c Spectrophotometer (Thermo Fisher Scientific, Waltham, MA, USA), and the samples were stored at −80 °C until use. The total RNA (1 µg) was reverse-transcribed using the iScript Select cDNA Synthesis Kit (Bio-Rad, Hercules, CA, USA) and real-time reverse transcriptase-polymerase chain reactions (RT-PCR) were performed, in duplicate for each sample, using the CFX Connect RT-PCR Detection System (Bio-Rad, Hercules, CA, USA), as previously described [19]. After amplification, a threshold was set for each gene, and Ct values were calculated for all of the samples. The gene expression changes were analyzed using the CFX Maestro 1.1 system software (Bio-Rad, Hercules, CA, USA). The primer sequences were designed using Beacon Designer software version 7.7 (Premier Biosoft International, Palo Alto, CA, USA), and were thoroughly tested. The forward (F) and reverse (R) primers used were as follows: F: GTTGAAGATATAATTGACACTG and R: GGCATATCCAACAACAAAC for *Hprt-1*; F: ACCTGTCCTGTGTAATGAAAG and R: GCTTGTGCTCTGCTTGTG for *Il-1β*; F: TTCCATCCAGTTGCCTTC and R: TTCTCATTTCCACGATTTCC for *Il-6*; F: GCTGTTAGAGACACTTCTGAG and R: CACTTTGGTAGGATTTGACTTTG for *Nos2*; F: CAAGGGACTAGCCAGGAG and R: TGCCTCTTCTGCCAGTTC for *Tnf-α*; and F: ATCAGACCTTCCTTGTAT and R: CACACTCATAGTTAAGACA for *Cox-2* (Eurofins Scientific, Luxembourg, Luxembourg). The results of at least three independent experiments were normalized using *Hprt-1* as a housekeeping gene, and were expressed as relative fold changes compared to the LPS, which was considered to be 100%.

### 2.6. Wound Healing Effect

The wound healing effect was assessed in NIH/3T3 cells using the scratch assay. Two horizontal lines were drawn at the bottom site of a 12-well plate as an aid for the microscopic imaging. The cells were seeded at a density of 1.75 × 10^5^ cells/well in 12-well plates, and were allowed to adhere for 24 h. A vertical artificial wound (scratch) was generated between the lines using a 20 µL pipette tip (0 h time point). The medium was discarded and the cells were incubated for 24 h with exposure medium (control), 0.16 mg/mL EO, or 0.8 μg/mL HRW. Both at the 0 and 24 h time points, four microscopic images of the scratch area were taken using a widefield microscope (Carl Zeiss, Oberkochen, Germany) at a magnification of 20×. The open wound area was determined by analyzing the images with the Fiji Is Just ImageJ software. The percentage of the wound closure in at least three independent experiments performed in duplicate was calculated by the following formula:

$$\%\;closed\;wound\;area=100\;\%-\left[\frac{open\;wound\;area\;24\;h}{open\;wound\;area\;0\;h}\right]\times 100$$

### 2.7. Anti-Senescent Effect 

#### 2.7.1. Senescence-Associated β-Galactosidase Activity

HaCaT and NIH/3T3 cells were seeded in 12-well plates at a density of 1.5 × 10^5^ or 2.5 × 10^4^ cells/well, respectively, and were allowed to adhere for 24 h. Thereafter, cellular senescence was induced with 100 µM etoposide (Sigma-Aldrich, St. Louis, MO, USA) for 72 h or 12.5 µM etoposide for 24 h in the HaCaT and NIH/3T3 cells, respectively. After the incubation period, the senescent cells and controls (exposed to etoposide-free medium) were treated in the absence or presence of 0.16 mg/mL EO or 0.8 μg/mL HRW for 24 h. Then, the culture medium was discarded, the cells were washed with PBS, fixed, and stained with freshly prepared β-galactosidase (β-gal) staining solution following the protocol provided by the manufacturer (Cell Signaling Technology, Danvers, MA, USA). Finally, the senescent cells were quantified using a widefield microscope (Carl Zeiss, Oberkochen, Germany) at a magnification of 40× by determining the percentage of β-gal-positive cells in randomly selected fields of four microscopic images, in at least three independent experiments performed in duplicate.

#### 2.7.2. Levels of the Senescence Marker p53 

HaCaT and NIH/3T3 cells were seeded in 6-well plates at a density of 6 × 10^5^ or 3 × 10^5^ cells/well, respectively. Senescence was induced for 24 h with etoposide (100 µM in HaCaT and 12.5 µM in NIH/3T3). Senescent and control cells were treated in the absence or presence of EO (0.16 mg/mL) or HRW (0.8 μg/mL) for 24 h. After the incubation period, the cells were scraped and lysed in ice-cold RIPA buffer [250 mM NaCl, 50 mM Tris, 1% (*v/v*) Nonidet P-40, 0.5% (*w/v*) sodium deoxycholate (DOC), 0.1% (*w/v*) sodium dodecyl sulfate (SDS), pH 8] supplemented with 1% (*v/v*) of the protease inhibitor cocktail (Sigma-Aldrich, St. Louis, MO, USA). The cell lysates were kept on ice for 20 min, then centrifuged at 18,000× *g* for 10 min at 4 °C, and the supernatants were collected and stored at −20 °C.

The protein concentration of the cell lysates was determined by the bicinchoninic acid protein assay (Thermo Fisher Scientific, Waltham, MA, USA). The samples were denatured for 5 min at 95 °C in 6× concentrated sample buffer [0.5 M Tris, 30% (*v/v*) glycerol, 10% (*w/v*) SDS, 0.6 M dithiothreitol (DTT), 0.012% bromofenol blue]. The cell lysates containing 35 μg protein were separated by electrophoresis in 12% (*w/v*) SDS polyacrylamide gels (SDS/PAGE). The proteins were then transferred to poly(vinylidene fluoride) (PVDF) membranes (Millipore, Burlington, MA, USA) in a suitable buffer (25 mM Tris, 192 mM glycine, 20% (*v/v*) methanol, pH 8.3) for 2 h at 750 mA. The membranes were blocked with 5% (*w/v*) BSA in Tris-buffered saline (150 mM NaCl, 25 mM Tris, pH 7.6) with 0.1% (*v/v*) Tween 20 (TBS-T) for 1 h at RT, and were incubated overnight at 4 °C with anti-p53 primary antibody (1:1000; #ab131442 Abcam, Cambridge, UK). After five washes with TBS-T, the membranes were incubated for 1 h at RT with the rabbit-specific secondary antibody (1:20,000; #31462, Invitrogen, Carlsbad, CA, USA). The protein bands were visualized by ECL chemiluminescence (Thermo Fisher Scientific, Waltham, MA, USA) on a ChemiDoc Imaging System (Bio-Rad, Hercules, CA, USA). Then, the lysate membranes were probed again with an anti-β-actin primary antibody (1:10,000; #A5441 Sigma-Aldrich, St. Louis, MO, USA) for the protein loading control. The bands were quantified with Image Lab software (Bio-Rad, Hercules, CA, USA). Results from at least three independent experiments were normalized to β-actin, and were expressed as the relative amount compared to the control.

#### 2.7.3. Metalloproteinase Activity Inhibition 

The cell-free assays for the evaluation of the inhibition of matrix metalloproteinase (MMPs) activity were performed in buffer containing 50 mM HEPES, 10 mM CaCl_2_ and 0.05% (*w/v*) Brij-35, pH 7.5. Briefly, in each well of a 96-well plate, 0.15 U/µL MMP-1, 0.009 U/µL MMP-9, or 0.013 U/µL MMP-13 enzymes (#BML-AK013, Enzo Life Sciences, Farmingdale, NY, USA) were pre-incubated with EO (0.16 mg/mL) or HRW (0.8 μg/mL) for 30 min at 37 °C, followed by the addition of 4 µM MMP fluorogenic substrate (#BML-P128, Enzo Life Sciences, Farmingdale, NY, USA). The fluorimeter was set to 37 °C, and the fluorescence was measured at 1-min time intervals for 30 min with a Fluorimeter SpectraMax Gemini EM (Molecular Devices, San Jose, CA, USA) at excitation and emission wavelengths of 340 nm and 440 nm, respectively. 

The effect of EO and HRW on MMP enzymatic activity was evaluated in at least three independent experiments, and the results were expressed as a percentage of the activity determined in the control conditions, which was considered to be 100%. EDTA—which is a chelating compound—at a concentration of 0.14 mM was used as the MMPs inhibitor (positive control) for each independent experiment.

#### 2.7.4. Expression of Collagen I

HaCaT cells were seeded in 6-well plates at a density of 6 × 10^5^ cells/well, and were allowed to adhere for 24 h. After this, the cells were incubated for 24 h with exposure medium (control) or treated with 0.16 mg/mL EO or 0.8 μg/mL HRW. The RNA extraction, cDNA synthesis and RT-PCR were performed as mentioned above. Forward (F) and reverse (R) primers used were as follows: F: GGAGGAGAGTCAGGAAAG and R: GCAACACAGTTACACAAGG for *Col1a1* (Eurofins Scientific, Luxembourg, Luxembourg). The results of at least three independent experiments were normalized using *Hprt-1* as a housekeeping gene, and were expressed as relative fold changes compared to the control.

### 2.8. Depigmenting Effect

B-16V cells were seeded in 6-well plates at a density of 6 × 10^5^ cells/well, and were allowed to adhere for 24 h. After that, the cells were incubated for 48 h, in triplicates, in exposure medium (control), or were treated with 200 µM 3-isobutyl-1-methylxanthine (IBMX) (Sigma-Aldrich, St. Louis, MO, USA), a well-known inducer of skin pigmentation, in the presence or absence of 0.16 mg/mL EO or 0.8 μg/mL HRW. At the end of the incubation period, the cells were washed twice with ice-cold PBS, and were then scraped and lysed in ice-cold lysis buffer [50 mM sodium phosphate (pH 6.5), 1% (*v/v*) Triton X-100, 0.1 mM phenylmethylsulfonyl fluoride (PMSF), 1 mM EDTA]. The cell lysates were kept at −80 °C for 20 min, defrosted, and then centrifuged at 12,000× *g* for 10 min at 4 °C; finally, the supernatants were collected for tyrosinase activity analysis. The protein content in the supernatants was determined using the bicinchoninic acid protein assay. The pellets were dissolved in 1 N NaOH for 1 h at 95 °C, transferred to a 96-well plate, and the absorbance was measured at 400 nm using the SpectraMax Plus 384 Spectrophotometer in order to determine their melanin content. The results of at least three independent experiments were expressed as the melanin content relative to the control, and were normalized by the protein concentration.

In order to determine the tyrosinase activity, the supernatants were transferred to a 96-well plate with 2.5 mM l-3,4-Dihydroxyphenylalanine (L-DOPA), a commercially available tyrosinase substrate (#D1507 Sigma-Aldrich, St. Louis, MO, USA). The tyrosinase activity was measured at 37 °C at 475 nm, at 5 min time intervals for 1 h, with a SpectraMax Plus 384 Spectrophotometer. The results of at least three independent experiments were expressed as the tyrosinase activity relative to the control, and were normalized according to their protein content.

Kojic acid (KA) (40 µM) (Sigma-Aldrich, St. Louis, MO, USA) was used as the positive control for each independent experiment.

### 2.9. Allergic Effect

#### 2.9.1. Expression of the Nrf2-Dependent Genes Hmox-1 and Nqo1

The increased expression of Nrf2-dependent genes in keratinocytes has been used to measure the skin sensitizing hazard [20]. As such, adapting OECD Test Guideline No. 442D for skin sensitization assessment [21], HaCaT cells were seeded in 6-well plates at a density of 6 × 10^5^ cells/well, and were allowed to adhere for 24 h. After that, the cells were incubated for 24 h with exposure medium (control), or were treated with 0.16 mg/mL EO or 0.8 μg/mL HRW. RNA extraction, cDNA synthesis and RT-PCR were performed as mentioned above. Forward (F) and reverse (R) primers used were as follows: F: CCTGAGTTTCAAGTATCC and R: AACAACAGAACACAACAA for *Hmox-1*; and F: GAGTCTGTTCTGGCTTAT and R: AACTGGAATATCACAAGGT for *Nqo1* (Eurofins Scientific, Luxembourg, Luxembourg). The results of at least three independent experiments were normalized using *Hprt-1* as a housekeeping gene, and were expressed as the relative fold changes compared to the control.

#### 2.9.2. Maturation of THP-1 Cells through the Up-regulation of the Co-stimulatory Molecules CD54 and CD86

The THP-1 cell line has also been used as a dendritic cell (DC) surrogate for the skin sensitizing hazard, in accordance with the OECD guidelines. As such, adapting the OECD Test Guideline No. 442E [22], THP-1 cells were stabilized overnight at a density of 5 × 10^5^ cells/mL. The next day, the cells were seeded in 12-well plates at density of 8 × 10^5^ cells/well, and were incubated for 24 h with medium (control), 0.16 mg/mL EO, or 0.8 μg/mL HRW. In addition, sensitization was induced with the strong skin allergen 1-fluoro-2,4-dinitrobenzene (DNFB) (8 µM) (Sigma-Aldrich, St. Louis, MO, USA) for 24 h. At the end of the incubation period, the cells were washed with ice-cold PBS supplemented with 1% (*v/v*) inactivated FBS, centrifuged at 300× *g* for 5 min at 4 °C, and the pellets were dissolved in ice-cold PBS supplemented with 1% (*v/v*) inactivated FBS. Then, 100 μL cell suspension was incubated with, or without, 3 μL anti-human CD54 antibody (#353111, Biolegend, San Diego, CA, USA) or anti-human CD86 (#305414, Biolegend, San Diego, CA, USA) antibody for 30 min at 4 °C. After that, the cells were washed, resuspended in ice-cold PBS supplemented with 1% (*v/v*) inactivated FBS, and analyzed using a flow cytometer (BD Accuri™ C6 Flow Cytometer, BD Biosciences, San Jose, CA, USA). A total of 1 × 10^4^ living cells were considered for the analysis. The CD86 and CD54 levels were analyzed by flow cytometry with the acquisition channel FL1 an FL4. Based on the geometric mean fluorescence intensity (MFI), the relative fluorescence intensity (RFI) of CD86 and CD54 for the positive control cells and chemical-treated cells was calculated according to the following equation:

$$RFI=\frac{MFI\;of\;chemical\;treated\;cells-MFI\;of\;chemical\;treated\;unstained\;cells}{MFI\;of\;control\;treated\;cells-MFI\;of\;control\;unstained\;cells}\times 100\;\;$$

Results from at least three independent experiments were expressed as a percentage of the RFI obtained in the control, which was considered to be 100%. If the RFI of CD54 and CD86 is equal to or greater than 200% and 150%, respectively, the samples are classified as skin sensitizers.

### 2.10. Skin Irritation

Skin irritation was evaluated using the SkinEthic™ Reconstructed Human Epidermis (RHE) model (EPISKIN Laboratories, Lyon, France), in compliance with the OECD Test Guideline No. 439 [23]. The RHE model consists of differentiated three-dimensional epidermal tissue of human keratinocytes grown on a 0.5 cm^2^ surface inert polycarbonate filter in a chemically defined medium at the air–liquid interface. Two specific media provided by EPISKIN were used: maintenance and growth media. On the day of receipt, the SkinEthic™ RHE inserts were transferred into 6-wells plates filled with 1 mL maintenance medium, and were stored in an incubator at 37 °C, under a 5% CO_2_–95% air atmosphere, for 24 h. For the experiments, SkinEthic™ RHE inserts were placed in maintenance medium in 24-well plates (300 µL/well), and EO (0.16 mg/mL) or HRW (0.8 μg/mL) was topically applied to the surface of the insert on three tissue replicates, for 42 min at RT. The experiment also included other inserts treated with PBS or 5% (*w/v* in water) SDS, which were used as the negative and positive controls, respectively. After the exposure time, the SkinEthic™ RHE inserts were rinsed 25 times with 1 mL PBS, and were transferred to 6-well plates with growth medium (2 mL/well) to be incubated for 42 h at 37 °C, under a 5% CO_2_–95% air atmosphere. The tissue viability was assessed using the MTT assay. For that, the tissues were incubated with MTT (1 mg/mL) at 37 °C for 3 h; then, the MTT solution was removed, and the formazan crystals were dissolved with isopropanol. The absorbance was measured at 570 nm using a SpectraMax Plus 384 Spectrophotometer. The results achieved on three tissue replicates were expressed as a percentage of the absorbance value obtained in the negative control, which was considered to be 100%, and were graphically presented as a percentage of the tissue viability. If the tissue viability value is <50%, the samples are classified as irritants.

### 2.11. Antifungal Activity

The antifungal activity of the EO and HRW was evaluated against several pathogenic strains: two clinical *Candida* strains isolated from recurrent cases of vulvovaginal and oral candidosis (*Candida krusei* H9 and *Candida guillermondii* MAT23), two *Candida* reference strains (*Candida albicans* ATCC 10231, Manassas, VA, USA and *Candida parapsilopsis* ATCC 90018, Manassas, VA, USA), one *Cryptococcus neoformans* reference (*C. neoformans* CECT 1078, Valencia, Spain), three dermatophyte clinical strains isolated from nails and skin (*Epidermophyton floccosum* FF9, *Microsporum canis* FF1, and *Trichophyton mentagrophytes* FF7), and four dermatophyte reference strains (*Microsporum gypseum* CECT 2908, Valencia, Spain; *Trichophyton mentagrophytes var. interdigitale* CECT 2958, Valencia, Spain; *Trichophyton rubrum* CECT 2794, Valencia, Spain and *Trichophyton verrucosum* CECT 2992, Valencia, Spain). All of the strains were subcultured in Sabouraud dextrose agar (SDA) or Potato dextrose agar (PDA) (Oxoid—Thermo Fisher Scientific, Waltham, MA, USA) before each test, in order to ensure optimal growth conditions and purity.

A macrodilution method was used to evaluate the minimal inhibitory concentrations (MICs) of the EO and HRW, according to the Clinical and Laboratory Standards Institute (CLSI) reference protocols M27-A3 [24] and M38-A2 [25] for yeasts and filamentous fungi, respectively. The inoculum suspensions were prepared from SDA or PDA cultures at appropriate densities in RPMI 1640 medium supplemented with 165 mM 3-(*N*-morpholino)propanesulfonic acid (MOPS) (Sigma-Aldrich, St. Louis, MO, USA), and were distributed into 12 × 75 mm glass test tubes. In order to obtain the concentrations of 0–10 mg/mL EO or 0–800 µg/mL HRW, serial twofold dilutions were prepared in DMSO for the EO or RPMI 1640 medium for the HRW, and were added to the inoculum suspensions in order to evaluate their concentration-response effect (the final DMSO concentrations never exceeded 2% *v/v*). EO or HRW-free positive controls and negative controls (PMI medium alone) were also included. The test tubes were incubated under aerobic conditions at 35 °C for 48 h/72 h for *Candida* spp./*Cryptococcus neoformans,* or at 30 °C for 7 days for dermatophytes. The MIC values were defined as the lowest concentration of the EO or HRW that caused complete growth inhibition. In order to investigate the minimal lethal concentrations (MLCs), 20 µL was removed from each negative tube—after the MIC determination—and cultured in SDA plates in the experimental conditions described above. The MLC values were defined as the lowest concentration of the EO or HRW that induced fungal death. At least three independent experiments were performed.

### 2.12. Statistical Analysis

The results are presented as the mean ± standard error of the mean (SEM) of the indicated number of experiments. The normality of the data distribution was assessed by the D’Agostino and Pearson and Shapiro–Wilk normality tests. Statistical comparisons between groups were performed by one-way analysis of variance (ANOVA) followed by Dunnett’s and Sidak’s multiple comparison tests. Significance was accepted at *p* values < 0.05. All of the statistical calculations were performed using GraphPad Prism software (8.0.2, GraphPad Software Inc., San Diego, CA, USA).

## 3. Results and Discussion

### 3.1. Chemical Characterization

#### 3.1.1. EO Composition

The EO was obtained with a yield of 1.7% (*v/w*, based on the weight of the fresh leaves). The EO yield is in accordance with previous studies performed using fresh leaves, and used the same extraction method [26,27,28]; one of them was conducted in Portugal [26,27,28]. The chemical characterization of the EO, which was carried out using GC-FID and GC-MS, is listed in Table 1; 1,8-cineole (72.3%) and α-pinene (9.4%) were found to be the main components. The composition of EOs can vary according to several factors, namely the method of distillation, the status of the leaves (fresh or dry), and the geographical region of their origin, as described previously [5]. Different amounts of 1,8-cineole in EOs extracted from *E. globulus* leaves were reported in studies carried out in different regions of Portugal, ranging from 36.7% to 74.6% [29,30,31]. A high 1,8-cineole content was also found in EOs obtained from leaves of *E. globulus* planted in other countries, namely Australia (90.0%) [32], Italy (95.5%) [33], and Argentina (98.9%) [34].

#### 3.1.2. HRW Phenolic Composition

**Figure 1:**
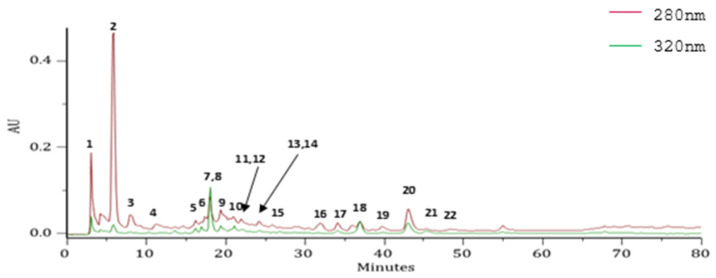
High-performance liquid chromatography with a photodiode array detector (HPLC-PDA) profile of the hydrodistillation residual water extract (HRW) from *E. globulus* leaves, recorded at 280 and 320 nm. AU, arbitrary units.

A yield of 11.4% (*w/w*, based on the weight of the fresh leaves) was obtained from the HRW of *E. globulus* leaves, in which polyphenols were identified using PDA spectra and MS^n^ data (Figure 1, Table 2). Gallic acid, 5-caffeoylquinic acid and ellagic acid were identified in HRW. Others polyphenols were also detected, such as ellagitannins, quercetin derivatives, and luteolin 7-*O*-glucuronide. Regarding the phenolic profiles of *E. globulus* leaves, the chemical composition is very heterogeneous, and can vary according to geographic region and the extractive solvent, as illustrated in several studies [7,10,13,35,36,37]. However, our results are in accordance with previous studies reporting phenolic acids as the predominant phenolic compounds in *E. globulus* leaves. Among the less abundant polyphenols, ellagitannins have been described prevail, which is in line with the results obtained for the HRW [7,10,12,13,15,35,36,37,38,39,40,41,42].

### 3.2. Cell Viability

**Figure 2:**
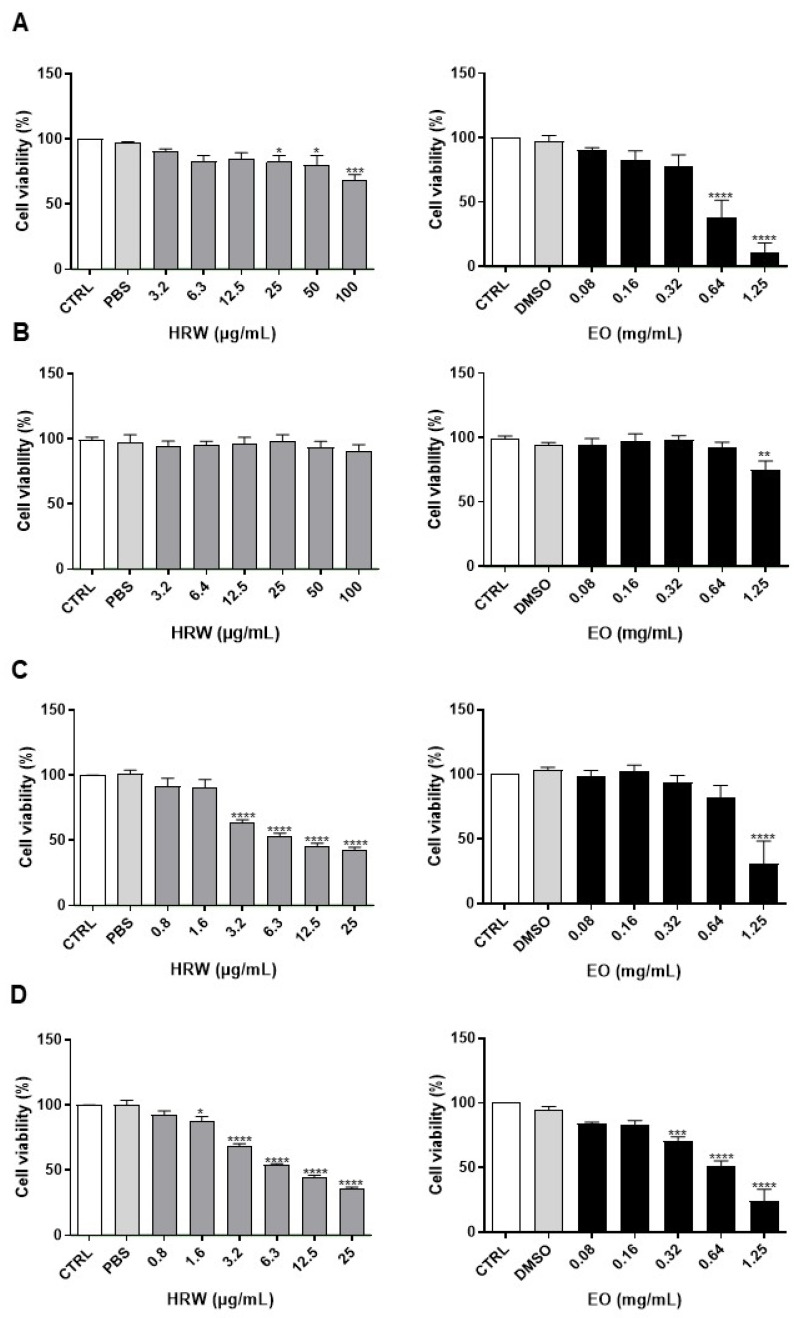
Effect of HRW and essential oil (EO) extracts from *E. globulus* leaves on the cell viability of RAW 264.7 macrophages (**A**), B-16V melanocytes (**B**), HaCaT keratinocytes (**C**), and NIH/3T3 fibroblasts (**D**). The cells were treated for 24 h with 0–1.25 mg/mL EO or 0–100 µg/mL HRW, and then their viability was evaluated using a 3-(4,5-dimethylthiazol-2-yl)-2,5-diphenyltetrazolium bromide (MTT) reduction assay. Cells treated with the medium alone were used as a control (CTRL), and cells treated with phosphate-buffered saline (PBS) or dimethyl sulphoxide (DMSO) were used as the solvent control. The results were expressed as the percentage (%) of cell viability relative to the CTRL, and represent the mean ± standard error of the mean (SEM) of at least three independent experiments performed in triplicate. The statistical analysis was carried out by one-way analysis of variance (ANOVA) followed by Dunnett’s multiple comparison test. * *p* < 0.05, ** *p* < 0.01, *** *p* < 0.001, and **** *p* < 0.0001: significantly different compared to the CTRL.

In order to evaluate the skin safety profile of EO and HRW extracts from *E. globulus* leaves, the cell viability was evaluated by the MTT assay in cells which are representative of the epidermis (keratinocytes and melanocytes) and dermis (macrophages and fibroblasts) (Figure 2). Regarding macrophages (Figure 2A), the absence of toxicity was observed after 24 h treatment with EO or HRW at concentrations below 0.32 mg/mL and 12.5 µg/mL, respectively, as cell viability was preserved. Regarding the effect of EO and HRW extracts on melanocytes (Figure 2B), no significant toxicity was observed after 24 h treatment with EO at concentrations below 0.64 mg/mL, and all of the tested concentrations of HRW were free of toxicity. In the case of keratinocytes (Figure 2C), non-toxic effects of EO were observed at 24 h for concentrations below 0.64 mg/mL, and similar results were obtained for concentrations of HRW below 1.6 µg/mL. Finally, an absence of toxicity was found in fibroblasts (Figure 2D) after 24 h of incubation at concentrations below 0.16 mg/mL for EO and 0.8 µg/mL for HRW. 

The cytotoxicity screening of EO and HRW extracts revealed safe concentrations in four mammalian cell lines representing skin cells (melanocytes, keratinocytes and fibroblasts) and dermal innate immune cells (macrophages) (Figure 2). Aaaza and co-authors reported that 0.5 mg/mL EO from *E. globulus* leaves decreases approximately 20% the viability of THP-1 cells [8], which is in accordance with our study showing 0.32 mg/mL EO to reduce the viability of RAW 264.7 macrophages by approximately 20%. However, Ahmed et al. found, in this cell line, an inhibition of 80% of cell viability induced by 0.26 µg/mL EO [43], revealing that the EO tested in this study is less toxic to these innate immune cells. The cytotoxicity of EO from *E. globulus* leaves in melanocytes, keratinocytes or fibroblasts has not been published previously, and the present study reveals, for the first time, safe concentrations of this extract in these representative skin cell lines. Regarding the HRW, two previous studies demonstrated that 50 µg/mL of a methanolic extract and 84 µg/mL of an ethanolic extract from *E. globulus* leaves are not toxic to murine bone marrow-derived macrophages and J774A.1 murine macrophages, respectively [10,11]. However, in the present study, the absence of toxicity was only observed with 12.5 µg/mL HRW in RAW 264.7 macrophages. Additionally, Park and co-authors demonstrated that 100 µg/mL of an ethanolic extract from *E. globulus* leaves has no toxicity in human dermal fibroblasts, while our study only revealed an absence of toxicity in NIH/3T3 fibroblasts for concentrations of the HRW below 0.8 µg/mL [15]. Cytotoxicity studies in keratinocytes or melanocytes using extracts obtained from *E. globulus* leaves have not been reported in the literature. *Eucalyptus* bioactive extracts are used in numerous cosmetic formulations; however, the safety of these cosmetics is not sufficiently supported in the literature [15]. Our study identified concentrations of EO and HRW extracts that are not deleterious to skin cells, thus encouraging the in-depth evaluation of their bioactive effects for the purposes of future incorporation in cosmetic and/or pharmaceutical formulations.

### 3.3. Anti-Inflammatory Effect

**Figure 3:**
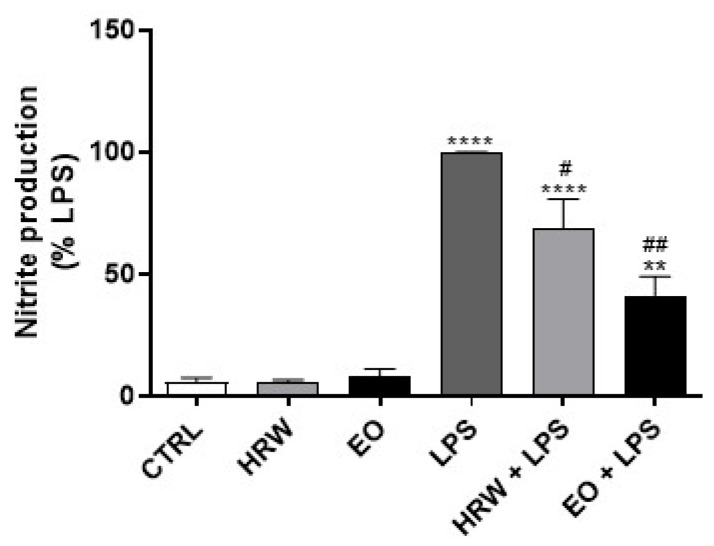
Effect of HRW and EO extracts from *E. globulus* leaves on nitric oxide (NO) production in lipopolysaccharide (LPS)-stimulated RAW 264.7 macrophages. The cells were treated with 0.32 mg/mL EO or 12.5 μg/mL HRW in the presence or absence of 1 μg/mL LPS, for 24 h. The cells treated with the medium alone were used as the CTRL. The NO production was determined in the cell culture supernatants using the Griess reagent. The results were expressed as the percentage (%) of nitrite production relative to the LPS; they represent the mean ± SEM of at least three independent experiments performed in triplicate. The statistical analysis was performed by one-way ANOVA followed by Dunnett’s and Sidak’s multiple comparison tests. ** *p* < 0.01 and **** *p* < 0.0001: significantly different compared to the CTRL. # *p* < 0.05 and ## *p* < 0.01: significantly different compared to the LPS.

The anti-inflammatory effect of the EO and HRW extracts from *E. globulus* leaves was assessed by determining their ability to inhibit the production of pro-inflammatory mediators in LPS-stimulated macrophages. Under these experimental conditions, LPS activates pro-inflammatory signaling pathways, namely the transcription factor NF-kB, which rapidly translocate into the nucleus to trigger the transcription of its target genes, such as *Nos2, Tnf-α, Il-6, Il-1β* and *Cox-2. Nos2* encodes the protein iNOS, which is responsible for the production of the pro-inflammatory mediator NO. The results obtained demonstrated that 0.32 mg/mL EO and 12.5 µg/mL HRW decrease NO levels by 58.85% and 31.11% in LPS-treated cells, respectively, in comparison with cells exposed to LPS alone (Figure 3). Dexamethasone and diclofenac are commonly used drugs for anti-inflammatory and analgesic purposes [44]. However, it is not possible to compare the anti-inflammatory activity of EO and HRW extracts—which are a mixture of several bioactive molecules—with those of pure chemicals such as dexamethasone or diclofenac, as the concentrations are not in the same range. Nevertheless, it is possible to compare the % of NO production of macrophages cultured in the presence of EO and HRW extracts with macrophages treated with the standard anti-inflammatory drugs, if the models are exactly the same. Although the experiments were not run in parallel, we have results addressing the anti-inflammatory effect of commercial anti-inflammatory drugs using the same model of inflammation addressed in this work, i.e., macrophages stimulated with LPS. For the concentration of 4 µg/mL dexamethasone, we achieved a decrease of approximately 70% of the NO (data not shown), which is a % similar to that obtained by EO (58.85%). The same was observed in a recent study in which 1.5 µg/mL diclofenac inhibited NO production by approximately 30%, which is comparable with the results obtained with HRW (31.11%) [45]. Although the concentrations in our study were higher to produce the same NO inhibition, these results show the strong anti-inflammatory effect of the studied extracts.

**Figure 4:**
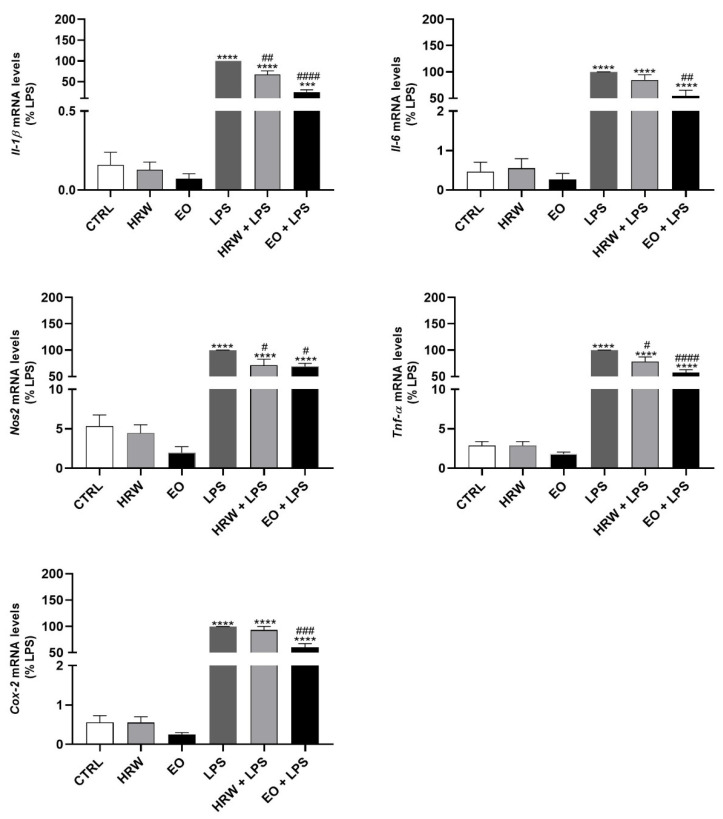
Effect of HRW and EO extracts from *E. globulus* leaves on the expression of pro-inflammatory genes in RAW 264.7 macrophages. The cells were pre-incubated for 18 h with 0.32 mg/mL EO or 12.5 μg/mL HRW, before incubation with or without 1 μg/mL LPS for an additional 6-h period. The cells treated with the medium alone were used as the CTRL. *Il-1β*, *Il-6*, *Nos2*, *Tnf-α* and *Cox-2* gene expression was evaluated by real-time reverse transcriptase-polymerase chain reactions (RT-PCR). The results, expressed as relative fold changes compared to the LPS, represent the mean ± SEM of at least three independent experiments. The statistical analysis was performed by one-way ANOVA followed by Dunnett’s and Sidak’s multiple comparison tests. *** *p* < 0.001 and **** *p* < 0.0001: significantly different compared to the CTRL. # *p* < 0.05, ## *p* < 0.01, ### *p* < 0.001, and #### *p* < 0.0001: significantly different compared to the LPS.

Additionally, the expression of pro-inflammatory genes that encode IL-1β, IL-6, iNOS, TNF-α and COX-2 was analyzed by RT-PCR (Figure 4). The EO strongly decreased the mRNA levels of *Il-1β*, *Il-6*, *Nos2*, *Tnf-α* and *Cox-2* triggered by LPS. Moreover, HRW also decreased the expression of *Il-1β*, *Il-6*, *Nos2*, *Tnf-α* in LPS-treated macrophages, and induced a slight—not significant—decrease in the *Cox-2* mRNA levels under similar conditions. These results suggest a potential anti-inflammatory effect of EO and HRW extracts from *E. globulus* leaves.

The described findings are supported by several in vitro and in vivo studies showing that EO from *E. globulus* leaves possesses strong anti-inflammatory activity. Aazza and co-authors demonstrated that this EO inhibits lipoxygenase on THP-1 cells, which was associated with the presence of limonene. However, the anti-inflammatory effect was higher in the presence of the EO than in the presence of limonene alone, suggesting that a synergistic effect between limonene and other EO components is responsible for this bioactivity [8]. On the other hand, Lin et al. reported that *E. globulus* EO exhibits anti-inflammatory effects in rats after swimming exercise, suggesting that EO-based aromatherapy during training can improve athletic performance [9]. Furthermore, *E. globulus* EO demonstrated anti-inflammatory action in chronic bronchitis induced by LPS in rats [46]. Silva and co-authors also reported that EO from *E. globulus* leaves exhibits anti-inflammatory effects, as demonstrated by the inhibition of rat paw edema induced by carrageenan and dextran, neutrophil migration into rat peritoneal cavities induced by carrageenan, and vascular permeability induced by carrageenan and histamine [47]. In these studies, the molecular mechanisms behind the EO-mediated anti-inflammatory effect are unclear. However, some investigators attributed the EO anti-inflammatory activity to the presence of monoterpenes, namely 1,8-cineole, which is a potent suppressor of cytokine release [48]. Nevertheless, it is important to take into consideration a study reporting that a high concentration of EO (hence, 1,8-cineole) affects the immune function in the respiratory tract and the overall organism immunity in rats, while a low concentration can have the opposite effect [49]. 

The anti-inflammatory potential of other extracts obtained from *E. globulus* leaves was also described in the literature. Two in vitro studies showed that ethanolic and methanolic extracts from *E. globulus* leaves decrease *Nos2, Il-1β* and *Tnf-α* mRNA levels and inhibit NO production in macrophages stimulated with LPS or LPS plus interferon-gamma (IFN-ϒ) [10,11]. Following these findings, a methanolic extract also reduced the levels of NO and pro-inflammatory mediators, as well as the regulatory transcription factor NF-kB, in mice administrated with cyclophosphamide [12]. These results might be attributable to the presence of phenolic compounds, as recent studies demonstrated their anti-inflammatory potential [50,51]. Interestingly, our results demonstrate that *E. globulus* leaves are a source of bioactive extracts, including the HRW described here for the first time, that exhibit a significant anti-inflammatory effect.

Inflammatory skin diseases are characterized by the activation of innate and adaptive immune responses via pro-inflammatory cytokine production [52]. The cutaneous immune response is crucial for skin aging regulation and the development of immune-mediated skin diseases, namely eczema, acne, atopic dermatitis, and psoriasis [53,54]. The common basis to treat these skin diseases is the control of inflammation; however, effective therapeutic agents are insufficient, and our results demonstrate that *E. globulus* extracts have enormous potential for inflammatory skin disease prevention and treatment.

In addition, our study reveals that EO and HRW extracts obtained from *E. globulus* have strong anti-inflammatory activity, providing scientific support for the traditional use of *E. globulus* biomass to relieve several conditions associated with deregulated responses to inflammation [55], and also for novel uses, as recently suggested by in silico analysis suggesting that EO from *E. globulus* can be utilized as a potential inhibitor against COVID-19 [56]. 

### 3.4. Wound Healing Effect

**Figure 5:**
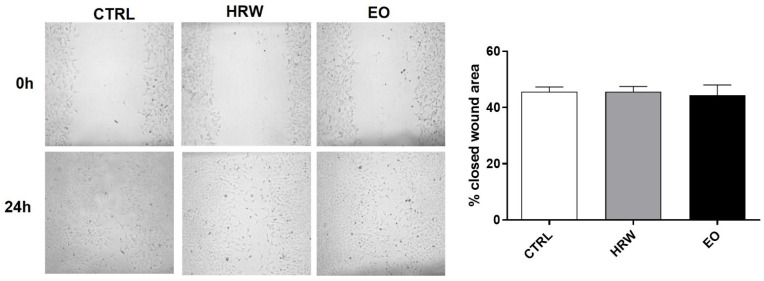
Wound healing effect of HRW and EO extracts from *E. globulus* leaves on NIH/3T3 fibroblasts. A mechanical injury was performed in cells incubated in the absence or presence of 0.16 mg/mL EO or 0.8 μg/mL HRW for 24 h. Cells treated with the medium alone were used as the CTRL. The closure of the wound area was determined by the analysis of the images with Fiji Is Just ImageJ software. The results—expressed as a percentage (%) of the closed wound area—represent the mean ± SEM of at least three independent experiments performed in duplicate. The statistical analysis was performed using one-way ANOVA followed by Dunnett’s multiple comparison test.

The wound healing effects of the EO and HRW extracts from *E. globulus* leaves were assessed in NIH/3T3 fibroblasts using the scratch assay (Figure 5). However, no significant differences were observed between the control and treated fibroblasts after 24 h. The same results were observed in fibroblasts treated with EO and HRW extracts for 12 h (data not shown). Although our in vitro study didn’t reveal differences in these skin cells exposed to EO and HRW extracts, two in vivo studies reported the healing effect of an EO isolated from the fruits of *E. globulus* [57], and of an ethanolic extract obtained from *E. globulus* leaves [58].

### 3.5. Anti-Senescent Effect

**Figure 6:**
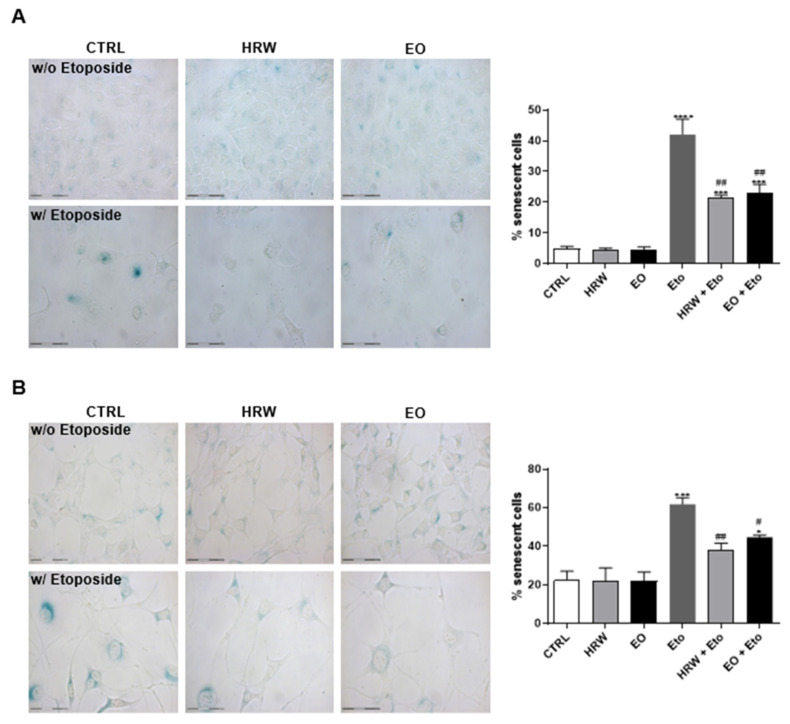
Effect of HRW and EO extracts from *E. globulus* leaves on the senescence-associated β-galactosidase activity in etoposide (Eto)-stimulated HaCaT keratinocytes (**A**) and NIH/3T3 fibroblasts (**B**). Cellular senescence was induced using 100 µM Eto for 72 h for HaCaT and 12.5 µM Eto for 24 h for NIH/3T3. After the incubation period, the senescent cells were treated in the absence or presence of 0.16 mg/mL EO or 0.8 μg/mL HRW for 24 h. Cells treated with the medium alone were used as the CTRL. The senescent cells were quantified using a Senescence β-Galactosidase staining kit. The results—expressed as the percentage (%) of the senescent cells—represent the mean ± SEM of at least three independent experiments performed in duplicate. The statistical analysis was performed using one-way ANOVA, followed by Dunnett’s and Sidak’s multiple comparison tests. * *p* < 0.05, *** *p* < 0.001, and **** *p* < 0.0001: significantly different compared to the CTRL. # *p* < 0.05, and ## *p* < 0.01: significantly different compared to the Eto.

**Figure 7:**
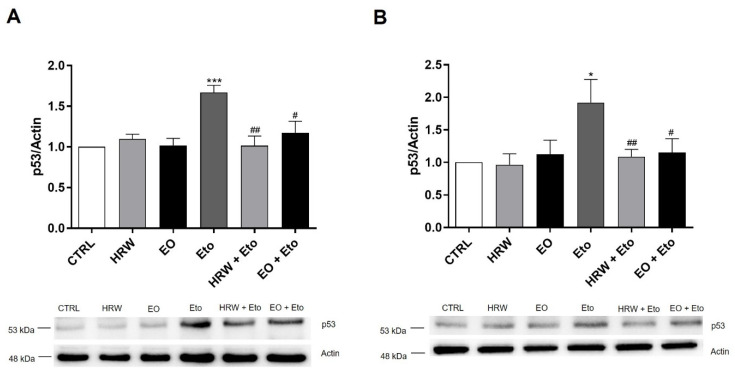
Effect of HRW and EO extracts from *E. globulus* leaves on levels of the p53 senescence marker in Eto-stimulated HaCaT keratinocytes (**A**) and NIH/3T3 fibroblasts (**B**). Cellular senescence was induced with 100 µM Eto during 72 h for HaCaT and 12.5 µM Eto during 24 h for NIH/3T3. After the incubation period, the senescent cells were treated in the absence or presence of 0.16 mg/mL EO or 0.8 μg/mL HRW for 24 h. Cells treated with the medium alone were used as the CTRL. The p53 levels were evaluated by Western Blot. The results normalized to β-actin and expressed as the relative amount compared to the CTRL, represent the mean ± SEM of at least three independent experiments. Statistical analysis was made by one-way ANOVA followed by Dunnett’s and Sidak’s multiple comparison tests. * *p* < 0.05, *** *p* < 0.001: significantly different compared to the CTRL. # *p* < 0.05, ## *p* < 0.01: significantly different compared to Eto.

The anti-senescent effects of the EO and HRW extracts from *E. globulus* leaves was evaluated using etoposide-stimulated HaCaT keratinocytes and NIH/3T3 fibroblasts with a commercially available kit (Figure 6). The etoposide-induced senescence in both keratinocytes and fibroblasts was indicated by the increase in the number of blue galactosidase-positive cells. It was shown that etoposide-induced senescence in keratinocytes was reduced in the presence of 0.16 mg/mL EO or 0.8 μg/mL HRW, by 19.01% and 20.52%, respectively (Figure 6A). Regarding fibroblasts, the same concentration of EO and HRW extracts reduced the % of senescent cells, in comparison with etoposide-treated cells, by 17.24% and 23.68%, respectively (Figure 6B). Under these conditions, the protein levels of p53 were evaluated by Western Blot (Figure 7). The upregulation of this senescence marker was induced by etoposide in comparison to the control cells, while treatment with 0.16 mg/mL EO or 0.8 μg/mL HRW decreased the p53 levels in the keratinocytes (Figure 7A) and fibroblasts (Figure 7B). 

**Figure 8:**
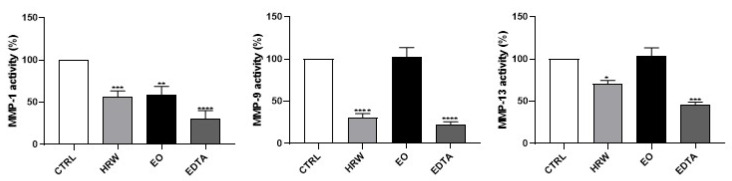
Effect of HRW and EO extracts from *E. globulus* leaves on MMP-1, MMP-9 and MPP-13 activity. In a cell-free system, MMP-1, MMP-9 or MMP-13 enzymes were pre-incubated with EO (0.16 mg/mL), HRW (0.8 μg/mL) or ethylenediamine tetraacetic acid (EDTA) (0.14 mM) for 30 min at 37 °C, followed by the addition of MMP fluorogenic substrate. The enzymatic activity results were expressed as the percentage (%) of enzyme inhibition relative to the activity determined in the CTRL; the results represent the mean ± SEM of at least three independent experiments. The statistical analysis was performed by one-way ANOVA followed by Dunnett’s multiple comparison test. * *p* < 0.05, ** *p* < 0.01, *** *p* < 0.001, and **** *p* < 0.0001: significantly different compared to the CTRL.

**Figure 9:**
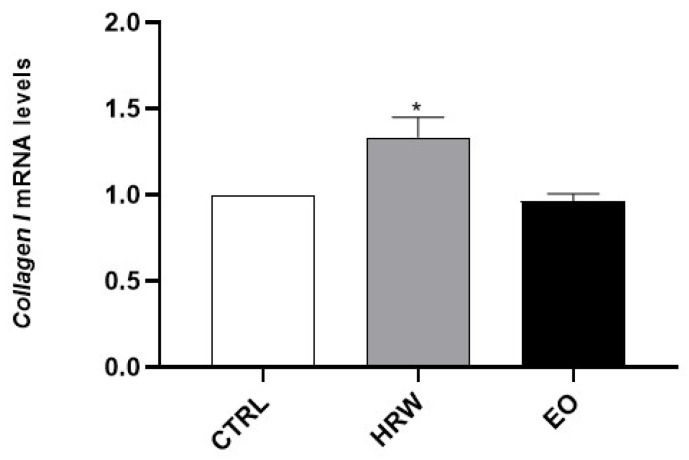
Effect of HRW and EO extracts from *E. globulus* leaves on the *collagen I* expression levels in HaCaT keratinocytes. The cells were treated in the absence or presence of 0.16 mg/mL EO or 0.8 μg/mL HRW for 24 h. Cells treated with the medium alone were used as the CTRL. The *Collagen I* gene expression was evaluated by RT-PCR. The results—expressed as relative fold changes compared to the CTRL—represent the mean ± SEM of at least three independent experiments. The statistical analysis was performed by one-way ANOVA, followed by Dunnett’s multiple comparison test. * *p* < 0.05: significantly different compared to the CTRL.

Next, the effects of EO and HRW extracts on MMP-1, MMP-9 and MPP-13 activity were evaluated (Figure 8). Furthermore, *collagen I* expression was analyzed in HaCaT keratinocytes upon exposure to these extracts (Figure 9). The results obtained demonstrated that EDTA, used as a positive control in the cell-free assay in order to evaluate the inhibition of MMPs, decreased all of the tested activities of MMPs. Similarly, 0.16 mg/mL EO and 0.8 μg/mL HRW decreased the MMP-1 activity by 43.24% and 41.16% compared to the control, respectively. On the other hand, at an HRW concentration of 0.8 μg/mL, the MMP-9 and MMP-13 activities were reduced by 69.29% and 29.25%, respectively. Additionally, the *collagen I* expression was significantly increased in keratinocytes treated with 0.8 μg/mL HRW.

The skin aging process is associated with alterations in the composition and structure of the dermal extra cellular matrix (ECM) [59]. The ECM is constituted of numerous proteins, including collagen and elastin, which have an important role in the maintenance of skin elasticity [60]. The MMPs—which are zinc-dependent collagenases—accumulate in aged skin, and its expression is also stimulated, resulting in collagen degradation [61]. Once collagen type 1 is the most important structural protein of ECM, its depletion is considered the principal cause of skin aging [61,62]. Additionally, senescent keratinocytes and fibroblasts appear to accumulate with age [63,64], and the accumulation of senescent cells plays a crucial role in the skin aging process, resulting in the loss of its function [65,66]. Enlarged and flattened cellular morphology, large nuclei, an irregular shape, the presence of cytoplasmic vacuoles, the accumulation of lipofuscin in lysosomes, increased ROS production, and the increased activity of the lysosomal Senescence-Associated β-galactosidase (SA-β-gal) are the principal characteristics of senescent cells [67,68,69,70]. In the present study, EO and HRW extracts decreased senescence induced by etoposide in keratinocytes and fibroblasts, suggesting its potential anti-senescence activity. The detection of SA-β-gal activity is frequently used as a first biomarker of senescence [71]. Nevertheless, further experiments with other senescence markers, such as p21 and p53—key effectors of cell cycle arrest leading to senescence—should be considered in order to confirm this activity. Accordingly, EO and HRW extracts decreased the p53 levels in etoposide-treated cells, thus confirming the anti-senescent effect. 

A study from Ishikawa et al. reported that treatment with *E. globulus* EO can be useful for the prevention of skin dryness by the up-regulation of the ceramide levels in dry skin [72]. Furthermore, UV-A and UV-B irradiation can also induce senescence in cells, and a recent study from Park and co-authors observed that the topical application of an ethanolic extract of *E. globulus* was able to reduce wrinkle formation, epidermal thickness, and collagen degradation in UVB-irradiated hairless mice. This study revealed that ethanolic extracts of *E. globulus* inhibit epidermal alteration and collagen collapse, increase skin hydration, and downregulate the proteins responsible for the aging process, namely MMP-1, elastin, procollagen type 1 and transforming growth factor-β (TGF-β), thus preventing UVB-induced skin photo damage [73]. The authors attributed this activity to the presence of gallic acid, which has been reported to protect skin from photoaging by regulating MMP-1 and TGF-β [74].

The HRW decreased the MMP-1, MMP-9 and MMP-13 activities, and upregulated the collagen type 1 in keratinocytes. Interesting, gallic acid is present in the HRW, suggesting that the anti-senescence effect of this extract could be due to the presence of gallic acid, as well as other phenolic compounds. In fact, several phenolic compounds have been investigated for their ability to prevent the development of a senescence phenotype in cells treated with various damaging agents [75]. For example, treatment with quercetin, which is also an HRW component, was shown to reduce the expression of IL-6, IL-8 and IL-1β and SA-β-galactosidase activity in senescent skin fibroblasts [76]. In addition, Chondrogianni and co-authors reported that quercetin exhibits rejuvenating effects by reducing SA-β-galactosidase activity, stimulating a younger morphology and proliferation rate in senescent fibroblasts when compared to non-senescent fibroblasts [77]. Regarding the other main compounds present in HRW, a study in hairless mice exposed to UV-B reported that the topical administration of ellagic acid displays photoprotective effects on skin wrinkle formation resulting from collagen depletion through the upregulation of MMP. In addition, this study demonstrated that ellagic acid prevents the inflammatory responses caused by UV-B [78]. Furthermore, a recent study with 5-caffeoylquinic acid showed its anti-aging effect in human fibroblasts and keratinocytes exposed to UV radiation. The 5-Caffeoylquinic acid decreased the MPP-1 levels, increased the collagen and hyaluronic acid content, and protected against the UV-induced increase in the pro-inflammatory cytokines IL-1β and IL-6, which are potential pathways of anti-aging effects [79]. Indeed, it has been demonstrated that immune cells are able to identify and eliminate senescent cells [80,81]. Nevertheless, the immune system decline associated with aging can be partially responsible for senescent cells’ accumulation in organisms with increased age [82]. Accordingly, the senescence of immune cells also has a role in declining immune functions [83]. 

Interestingly, several bioactive compounds can induce senescence in healthy cells; however, it was demonstrated here that EO and HRW extracts don’t have senescent effects per se in fibroblasts and keratinocytes. Senescent cells accumulate in tissues during aging, and emerge in the altered tissues of patients with age-associated diseases [84]. Here, we demonstrate that EO and HRW extracts have the ability to protect keratinocyte and fibroblast skin cells against etoposide-induced senescence. Specifically, the HRW was found to inhibit metalloproteinase activity, and to upregulate collagen. Taken together, these data suggest that *E. globulus* biomass is a promising source of bioactive ingredients to be incorporated in cosmetic and skincare products for the prevention and/or attenuation of skin aging and age-related conditions.

### 3.6. Depigmenting Effect

**Figure 10:**
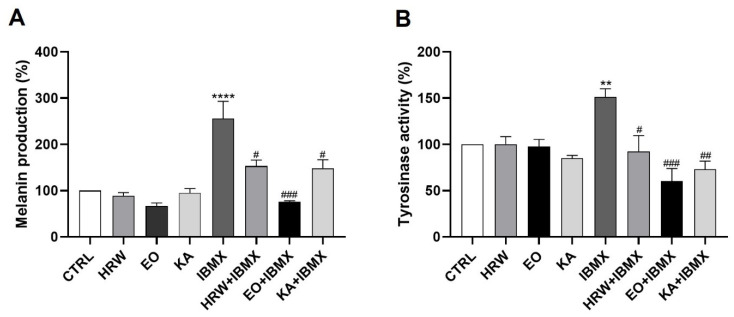
Effect of HRW and EO extracts from *E. globulus* leaves on the melanin production (**A**) and tyrosinase activity (**B**) in 3-isobutyl-1-methylxanthine (IBMX)-stimulated B-16V melanocytes. The cells were treated, in the absence or presence of 0.16 mg/mL EO, 0.8 μg/mL HRW or 40 µM kojic acid (KA) (positive control) for 72 h, with 200 µM IBMX, a melanogenesis inducer. Cells treated with the medium alone were used as the CTRL. The tyrosinase activity and melanin content were determined by a spectrophotometric method. The results were expressed as a percentage (%) of the CTRL, and represent the mean ± SEM of at least three independent experiments. The statistical analysis was performed by one-way ANOVA followed by Dunnett’s and Sidak’s multiple comparison tests. ** *p* < 0.01 and **** *p* < 0.0001: significantly different compared to the CTRL. # *p* < 0.05, ## *p* < 0.01, and ### *p* < 0.001: significantly different compared to the IBMX.

The depigmenting effect of EO and HRW extracts from *E. globulus* leaves was evaluated on IBMX-stimulated B-16V melanocytes through the analysis of their tyrosinase activity and the quantification of their melanin content (Figure 10). The production of melanin, the pigment responsible for skin pigmentation, was increased after IBMX treatment by 156.14% relative to the control, and was reduced in the presence of 0.16 mg/mL EO or 0.8 μg/mL HRW by 180.73% and 103.03%, respectively (Figure 10A). Tyrosinase plays a key role in melanogenesis, IBMX increased the tyrosinase activity by 51.19% relative to the control, and the above concentrations of EO and HRW extracts were shown to inhibit this enzyme in IBMX-treated cells by 90.69% and 58.82%, respectively (Figure 10B). The melanin production and tyrosinase activity were also reduced by KA, which is a depigmenting agent used in skin products, by 107.64% and 77.92%, respectively. The concentration of EO used was higher than that of KA (40 μM and 5.7 μg/mL); however the melanin and enzyme inhibition effect of EO was higher than that of KA. On the other hand, a lower concentration of HRW demonstrated similar effects to KA, showing its potential as a depigmenting agent.

Several skin pigmentation disorders—such as freckles, melasma, age spots, post-inflammatory melanoderma and others—affect the color of the skin, and result from the abnormal accumulation of melanin, which is a pigment made by specialized cells in the skin, the melanocytes [85,86]. Tyrosinase is the rate-limiting enzyme of melanin production, and many tyrosinase inhibitors, such as KA [87], are used in skin whitening products in order to prevent or treat abnormal skin pigmentation [88]. Our results indicated that EO and HRW extracts exhibit a potent depigmenting effect by inhibiting intracellular tyrosinase activity, and subsequently by decreasing melanin production. Accordingly, a previous study also revealed that eucalyptus flower EO suppresses tyrosinase activity and depletes melanin in B16F10 mouse melanoma cells [89]. This study indicated that the depigmenting effect occurs due to the EO antioxidant properties, and by the down-regulation of both mitogen-activated protein kinases (MAPK) and protein kinase A (PKA). Furthermore, Hasegawa et al. described the depigmenting effect of a monoterpene glycoside conjugated with gallic acid from the leaves of *E. globulus* by decreasing melanin synthesis in cultured murine B16F1 melanoma cells [90].

However, it has been reported that whitening products used as skin depigmenting agents have several side effects [91,92,93], including contact dermatitis caused by KA [91]. As such, the development of an effective and safe skin depigmenting agent is necessary in the cosmetic field. In fact, the findings obtained in this study indicate that the EO and HRW extracts have the potential to be developed as safe and effective skin depigmenting agents, as these extracts don’t cause allergic contact dermatitis or skin irritation.

### 3.7. Allergic Effect

**Figure 11:**
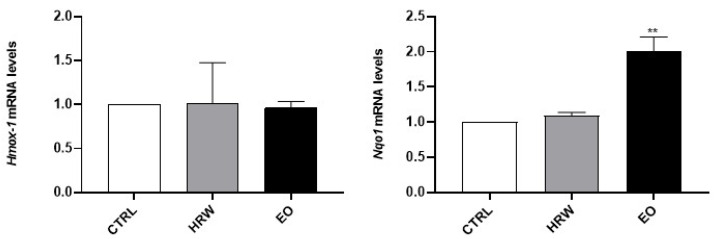
Effect of HRW and EO extracts from E. globulus leaves on the *Hmox-1* and *Nqo1* expression levels in HaCaT keratinocytes. The cells were treated in the absence or presence of 0.16 mg/mL EO or 0.8 μg/mL HRW for 24 h, and then the *Hmox-1* and *Nqo1* gene expression was evaluated by RT-PCR. The cells treated with the medium alone were used as the CTRL. The results—expressed as relative fold changes compared to the CTRL—represent the mean ± SEM of at least three independent experiments. The statistical analysis was performed by one-way ANOVA followed by Dunnett’s multiple comparison test. ** *p* < 0.01: significantly different compared to the CTRL.

The requirement for non-animal alternatives has been recognized for the screening of chemicals’ hazard to human health in general, but it has become particularly pressing for cosmetic ingredients due to the full implementation of testing and marketing bans on animal testing under the European Cosmetics Regulation. Fortunately, for some specific endpoints, such as skin irritation and skin sensitization, validated alternatives are already available to be performed during new product development, and were used here to assure the safety profile of EO and HRW extracts from *E. globulus* leaves. Therefore, following the OECD guidelines for skin sensitization assessment, the increased expression of Nrf2-dependent genes in keratinocytes (namely *Hmox-1* and *Nqo1*) and the up-regulation of the co-stimulatory molecules CD54 and CD86 in THP-1 cells were used in this work to discard the potential of EO and HRW extracts to trigger skin sensitization. Regarding the expression of Nrf2-dependent genes (Figure 11), the results demonstrated that EO did not modulate the expression of *Hmox-1*, although a significant increase in *Nqo1* mRNA levels was noticed. Moreover, the HRW didn’t modulate *Hmox-1 and Nqo1* expression in these skin cells. The upregulation of *Hmox-1* and *Nqo1* genes has been reported for skin contact sensitizers [94]; therefore, these results suggest that the HRW doesn’t induce skin sensitization, but the results obtained with EO need to be further expanded to other Nrf2-dependent genes, as EO increased the expression of one of the two markers evaluated. 

**Figure 12:**
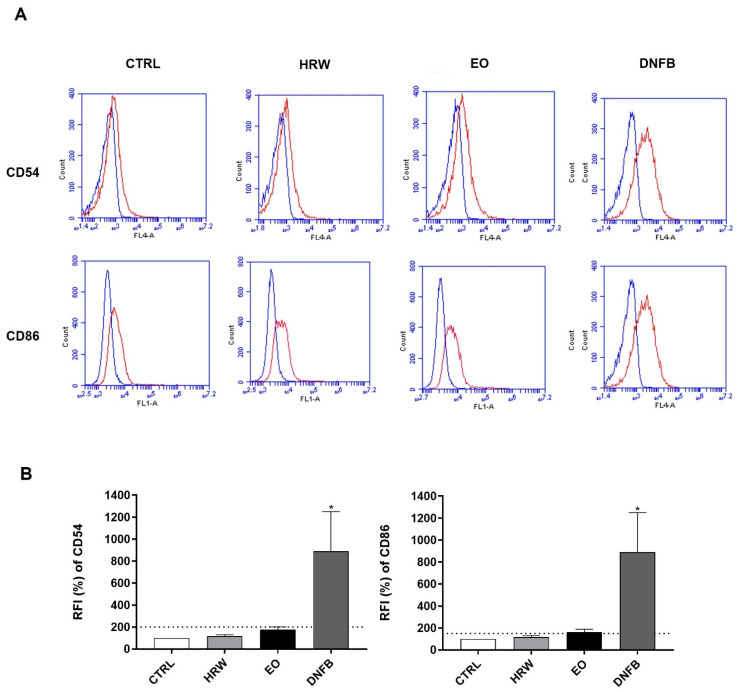
Phenotypic characterization (**A**) and effect on the levels of the co-stimulatory proteins CD54 and CD86 (**B**) of HRW and EO from *E. globulus* leaves in THP-1 monocytes. The cells were treated in the absence or presence of 0.16 mg/mL EO or 0.8 μg/mL HRW for 24 h. In addition, skin sensitization was induced with 8 µM 1-fluoro-2,4-dinitrobenzene (DNFB) for 24 h. The cells treated with the medium alone were used as the CTRL. The blue line represents unstained cells, and the red line represents the specific levels of the tested cells. The levels of CD54 and CD86 were measured by flow cytometry. The results—expressed as a percentage (%) of the fluorescence intensity (RFI) relative to the CTRL—represent the mean ± SEM of at least three independent experiments. The statistical analysis was performed by one-way ANOVA, followed by Dunnett’s and Sidak’s multiple comparison tests. * *p* < 0.05: significantly different compared to the CTRL.

In order to further corroborate the absence of skin sensitization potential attributable to EO and HRW extracts, changes in CD54 and/or CD86 co-stimulatory protein levels were evaluated by flow cytometry after the treatment of THP-1 cells (a dendritic cell surrogate) with 0.16 mg/mL EO or 0.8 μg/mL HRW (Figure 12). Indeed, one of the key events associated with skin sensitization is the maturation of dendritic cells, as assessed by the quantification of the surface expression of CD86 and CD54 proteins. As expected, treatment with DNFB, which is a strong skin sensitizer, increased the levels of CD54 and CD86. In contrast, EO and HRW extracts did not modify the levels of the co-stimulatory molecules CD54 and CD86, presenting RFI of CD54 and CD86 lower than 200% and 150%, respectively, which are thresholds used to classify a substance as a skin sensitizer.

Taken together, these results indicate that both *E. globulus* extracts do not trigger skin sensitization, thus corroborating their potential to be incorporated in cosmetic and skincare products.

### 3.8. Skin Irritation 

**Figure 13:**
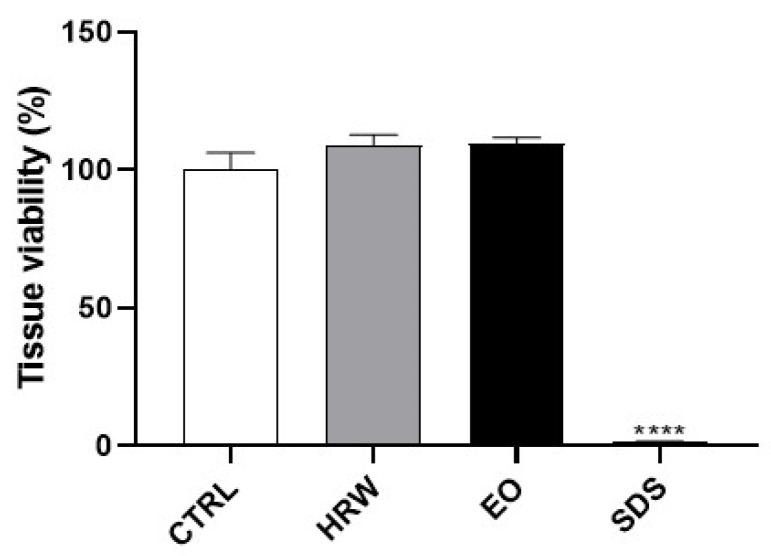
Effect of HRW and EO extracts from *E. globulus* leaves on tissue viability in Reconstructed Human Epidermis (RHE). The inserts were treated in the absence or presence of 0.16 mg/mL EO, 0.8 μg/mL HRW, or 5% sodium dodecyl sulfate (SDS) (positive control) for 42 min. Cells treated with the medium alone were used as the CTRL. The tissue viability was assessed by the MTT assay. The results were expressed as a percentage (%) of the tissue viability relative to the CTRL, and represent the mean ± SEM of at least three independent experiments. The statistical analysis was performed by one-way ANOVA, followed by Dunnett’s multiple comparison test. **** *p* < 0.0001: significantly different compared to the CTRL.

We further used the SkinEthic™ Reconstructed Human Epidermis model in compliance with OECD Test Guideline No. 439, aiming to address the potential of EO and HRW extracts from *E. globulus* leaves to evoke skin irritation (Figure 13). The results demonstrate that, as expected, the positive control (SDS) decreased the tissue viability. In contrast, the EO and HRW extracts did not show any irritant activity, presenting a tissue viability higher than 50%, which is a threshold used to classify a substance as non-irritant according to ISO 10993-10: 2010.

### 3.9. Antifungal Effect

The EO and HRW extracts were used to evaluate the antifungal activity of *E. globulus* biomass against several pathogenic strains involved in skin human diseases, and the results obtained are presented in Table 3. In general, the EO demonstrated modest antifungal activity against all of the tested strains, with MICs ranging from 1.25 to 5 mg/mL. Previous studies have reported the antifungal effect of EO from *E. globulus* leaves against *Candida albicans*. Vratnica et al. demonstrated that *E. globulus* EO has a high potency against *Candida albicans*, which was two times more effective than nystatin, a standard drug used to treat fungal infections of the skin, mouth, vagina, and intestinal tract [95]. However, Tyagi and Malik reported a higher MIC value for *Candida albicans* [96], demonstrating variability between the studies. Nevertheless, it has been reported before that *E. globulus*-derived EOs can inhibit the growth of some fungal species. The present study evaluated, for the first time, the antifungal activity of EO against other *Candida* species and *Cryptococcus neoformans*. Likewise, Nardoni et al. demonstrated that *E. globulus* EO is efficient against dermatophytes such as *Microsporum canis*, *Microsporum gypseum*, *Trichophyton mentagrophytes*, *Trichophyton terrestre* and *Trichophyton erinacei* [97]. In our study, the low activity of the EO against dermatophytes was observed, but besides this, the EO activity against *Epidermophyton floccosum*, *Trichophyton mentagrophytes var. interdigitale*, *Trichophyton rubrum* and *Trichophyton verrucosum* was evaluated for the first time in this study. 

The HRW had no inhibitory effect on the growth of the tested *Candidas* spp. strains even at the maximum concentration used. However, Bakht and co-authors concluded that *Candida albicans* was moderately susceptible to an aqueous extract from *E. globulus* leaves, as well as n-butanol and crude methanolic extracts [98]. On the other hand, our results demonstrate the moderate antifungal activity of HRW against dermatophytes, particularly for *Microsporum canis* and *Epidermophyton floccosum,* with MIC and MLC values of 200 µg/mL. To our knowledge, there is only one study in the literature regarding the antifungal activity of *E. globulus* leaf extracts against dermatophytes. Takahashi and co-authors reported that a methanol-dichloromethane extract prepared from *E. globulus* leaves exhibited antifungal activity, namely against *Trichophyton mentagrophytes* [42], which was attributed to the presence of three flavonoids, specifically 2′,6′-dihydroxy-3′-methyl-4′-methoxy-dihydrochalcone, eucalyptin, and 8-desmethyl-eucalyptin.

## 4. Conclusions

In conclusion, the present study described, for the first time, a bioactive extract obtained from the residual water from the hydrodistillation of fresh *E. globulus* leaves to obtain the EO, which we called HRW. It was also demonstrated that EO and HRW extracts attenuate the senescence/aging and the melanogenic phenotype in skin cells, and that they simultaneously exhibit an anti-inflammatory profile in immune cells. In addition, bioactive concentrations of both extracts were found to be free of cytotoxicity, supporting their future exploitation for oral or topical application. Finally, this study revealed that EO and HRW extracts present a safe profile for skin aesthetic purposes, as no skin irritation or sensitization were detected. Taken together, these findings suggest that *E. globulus* biomass, in particular the leaves, is a relevant source of bioactive substances are able to be incorporated in cosmetic and pharmaceutical products.

## 5. Patents

This work has two Portuguese pending patents (references 117531 and 117613).

## Figures and Tables

**Table 1:** Chemical characterization of the essential oil (EO) from *E. globulus* leaves by gas chromatography (GC-FID) and gas chromatography coupled to mass spectrometry (GC-MS).

RI^a^	RI^b^	Compound *	%
928	1025	α-Pinene	9.4
940	1077	Camphene	0.1
944	1131	Verbenene	t
967	1113	β-Pinene	0.2
978	1156	Myrcene	0.1
994	1167	α-Phellandrene	t
1010	1269	*p*-Cymene	0.8
1017	1201	Limonene	2.3
1017	1213	1,8-Cineole	72.3
1044	1248	γ-Terpinene	0.2
1056	1438	*cis*-Linalool oxide	t
1066	1466	*trans*-Linalool oxide	t
1068	1439	Cymenene	0.1
1074	1291	Terpinolene	0.1
1096	1574	Fenchyl alcohol	0.2
1103	1489	α-Campholenal	0.2
1118	1645	*E*-Pinocarveol	3.6
1133	1563	Pinocarvone	1.4
1143	1720	Mentha-1,5-dien-8-ol	0.3
1143	1698	Borneol	0.3
1156	1594	Terpinene-4-ol	0.3
1156	1842	*p*-Cymene-8-ol	0.1
1163	1879	*Z-p*-mentha-1(7),8 diene-2-ol	0.6
1167	1690	α-Terpineol	0.9
1177	1788	Myrtenol	0.1
1193	1828	*trans*-Carveol	0.2
1199	1879	*E-p*-menth-1(7)8-dien-2-ol	0.6
1212	1731	Carvone	0.1
1264	1574	Bornyl acetate	t
1326	1690	α-Terpinyl acetate	1.2
1403	1591	*E*-Caryophyllene	0.1
1424	1602	Aromadendrene	0.2
1443	1663	α-Humulene	0.1
1447	1636	Alloaromadendrene	t
1478	1720	δ- Selinene	t
1548	1916	Palustrol	t
1554	2110	Spathulenol	t
1557	1971	Caryophyllene oxide	t
1558	2062	Globulol	1.6
1568	2064	Viridiflorol	0.1
1594	2093	10-*epi*-γ-Eudesmol	0.2
1617	2188	α-Muurolol	0.1
1623	2215	β-Eudesmol	0.1
1629	2218	α-Cadinol	t
**Total identified**			**98.7**

* Compounds listed in order of their elution on the SPB-1 column. t: traces (≤ 0.05%); RI^a^: retention indices on the SPB-1 column relative to C8 to C23 *n*-alkanes; RI^b^: retention indices on the SupelcoWax-10 column relative to C8 to C23 *n*-alkanes.

**Table 2:** Chemical characterization of the hydrodistillation residual water extract (HRW) from *E. globulus* leaves by high-performance liquid chromatography with a photodiode array detector coupled to mass spectrometry with electrospray ionization (HPLC-PDA-ESI/MS^n^).

Peak *	R_t_ (min)	λ_max_ (nm)	HPLC-ESI-MS^n^ [*m/z* (Relative Abundance, %)]			Attempt to Identify
			Precursor Ion [M-H]	MS^2^	MS^3^	
1	3.24	234sh, 238, 246, 257	633(100)	301(100), 275 (16), 249 (15)	284 (34), 257 (80), 229 (100), 201 (15), 185 (55), 173 (13)	HHDP galloyllglucose
2	4.38	234sh, 238, 246, 270	169(100)	125(100)	125 (46), 107 (13), 97(100), 81 (96), 79 (28), 69 (12)	Gallic acid
3	5.63	231sh, 234sh, 238, 246, 277	633(100)	301(100)	284 (61), 257(100), 229 (63), 213 (11), 201 (31), 185 (70)	HHDP galloyllglucose
4	12.39	231sh, 238, 246, 265	783(100)	935 (24), 765(100), 613 (12)	721 (12), 613 (90), 597(100), 533 (13), 443 (11), 427 (29), 401 (20), 399 (17), 325 (19), 301 (58), 275 (63), 273(15), 231 (15), 229 (12)	bis-HHDP-glucose
5	17.14	231sh, 238, 246sh, 256, 289sh	353(100)	233(100)	205(100)	3-Caffeoyl-quinic acid
6	18.36	229sh, 238, 246sh, 252, 288sh, 299, 325	431(100)	385(100), 223 (16)	295 (11), 223(100), 205 (57), 161 (48), 153 (57), 151 (12)	Sinapoyl-hexoside
7	18.93	-	191(100)	173 (90), 127(100), 85(65)	109(100), 85 (55)	Quinic acid
8	18.93	231sh, 238, 246sh, 252, 292sh, 299sh, 325	353(100)	191(100)	127(100), 172 (60), 85(55)	5-Caffeoyl-quinic acid
9	20.03	232sh, 238, 246, 259, 330sh	461(100)	415(100)	269(100), 247 (14), 161 (33)	Not identified
10	21.13	234sh, 238, 246, 259, 368sh	1085(100)	783 (12), 765(100)	613 (58), 597 (61), 595 (14), 427 (21), 399 (27), 383 (19), 301(100), 275 (51), 273 (14), 259 (10), 231 (14), 229 (21)	Cornusiin B or eucalbanin A
11	21.52	232sh, 238, 246, 259, 371sh	1085(100)	1069 (25), 765(100), 755 (30), 451 (12)	-	Cornusiin B or eucalbanin A
12	22.10	226sh, 234sh, 238, 246, 258sh, 265, 366sh	451(100)	313 (40), 271 (84), 211(100), 169 (92), 151 (13)	168(100), 124 (12)	Galloyl-glucose ester
13	22.68	233sh, 238, 246, 261, 299sh, 358sh	1253(100)	1074 (47), 971(100), 781 (39), 640 (79)	-	Punicalin derivative
14	25.25	233sh, 238, 246, 257, 269sh, 309sh, 353sh, 362, 367sh	565(100)	550 (78), 549 (12), 519 (17), 419(100), 405 (60). 401 (44), 386 (28), 373 (21), 355 (11), 233 (18), 202 (21), 187 (19)	404(100), 373 (25)	Not identified
15	26.52	231sh, 234sh, 238, 246, 262, 292sh, 352sh	275(100)	257(100), 247 (13), 231 (14), 229 (31), 203 (21)	-	Not identified
16	34.34	226sh, 234sh, 238, 243sh, 246sh, 258sh, 268, 356sh	497(100)	331(100), 169 (79)	169(100), 125 (22)	Eucaglobulin
17	35.48	231sh, 234sh, 238, 246sh, 255, 265sh, 346sh, 364, 380sh	477(100)	315(100), 300 (20)	300(100)	Methylellagic acid hexose
18	38.16	232sh, 234sh, 238, 246, 258, 265sh, 354, 381sh	477(100)	301(100)	273 (15), 257 (12), 179(100), 151 (85)	Quercetin-*O*-glucuronide
19	39.20	227sh, 238, 239sh, 242sh, 244sh, 246sh, 260, 266sh, 295sh, 354, 381sh	609(100)	301(100), 271 (35), 255 (12)	273 (12), 239 (13), 179(100), 151 (79)	Quercetin 3-*O*-rutinoside
20	43.08	234sh, 238, 246sh, 255, 267sh, 306sh, 351sh, 367, 382sh	301(100)	284 (55), 257 (84), 245 (13), 229(100), 201 (22), 185 (78)	212 (13), 201 (69), 185(100), 173 (34), 157 (34), 145 (34)	Ellagic acid
21	45.25	229sh, 232sh, 238, 241sh, 246sh, 257, 267sh, 301sh, 351, 381sh	447(100)	301(100)	273 (19), 179(100), 151 (81)	Quercetin3-*O*-rhamnoside
22	46.94	231sh, 234sh, 238, 242sh, 246sh, 257sh, 261, 346 267sh, 293sh, 357sh, 380sh, 412sh, 420sh, 446sh, 466sh, 486	461(100)	285(100), 173 (14)	-	Luteolin 7-*O*-glucuronide

* The number of the peaks in this table corresponds to the peaks indicated in Figure 1. MS^2^ = 1st generation product ion spectra; MS^3^ = 2nd generation product ion spectra.

**Table 3:** Antifungal effect of *E. globulus* leaf EO and HRW extracts for *Candida* spp., *Cryptococcus neoformans* and dermatophytes.

Strains	EO		HRW	
	MIC ^a^	MLC ^a^	MIC ^b^	MLC ^b^
*Candida albicans*	5	5	>800	>800
*Candida krusei*	5	5	>800	>800
*Candida guilliermondii*	2.5	5	>800	>800
*Candida parapsilosis*	5	5	>800	>800
*Cryptococcus neoformans*	2.5	5	400	>800
*Trichophyton mentagrophytes*	2.5	2.5	400	800
*Trichophyton rubrum*	2.5	2.5	400	400
*Trichophyton mentagrophytes var. interdigitale*	5	5	400	800
*Trichophyton verrucosum*	2.5	2.5	>800	>800
*Microsporum gypseum*	5	5	800	>800
*Microsporum canis*	2.5	2.5	200	200
*Epidermophyton floccosum*	1.25	2.5	200	200

The minimal inhibitory concentration (MIC) and minimal lethal concentration (MLC) were determined by a macrodilution method, and are expressed in ^a^ mg/mL and in ^b^ µg/mL.

## Data Availability

All data are available upon contact of corresponding author.
